# Self-Medication Related Knowledge, Attitudes, and Practices among Residents of Riyadh, Saudi Arabia: A Community-Based Cross-Sectional Study

**DOI:** 10.3390/healthcare11233040

**Published:** 2023-11-25

**Authors:** Awad Mohammed Al-Qahtani, Nasser Saeed Alqahtani, Basheerahmed Abdulaziz Mannasaheb, Eisa Yazeed Ghazwani, Ibrahim Ahmed Shaikh, Bayan Fuad Abbag, Mamdouh Saleh Alharbi, Syed Arif Hussain

**Affiliations:** 1 Department of Family and Community Medicine, College of Medicine, Najran University, Najran 66462, Saudi Arabia; awadresearch17@gmail.com (A.M.A.-Q.); drnasser1000@hotmail.com (N.S.A.); eyghazwani@nu.edu.sa (E.Y.G.); bfabbag@nu.edu.sa (B.F.A.); 2 Department of Pharmacy Practice, College of Pharmacy, AlMaarefa University, Diriyah, Riyadh 13713, Saudi Arabia; 3 Department of Pharmacology, College of Pharmacy, Najran University, Najran 66462, Saudi Arabia; i.ibrahimshaikh09@gmail.com; 4 Respiratory Care Department, College of Applied Sciences, AlMaarefa University, Diriyah, Riyadh 13713, Saudi Arabia

**Keywords:** self-medication, Riyadh, community, knowledge, attitude, practice, world health organization, United Nations, sustainable development goals

## Abstract

Rational and responsible self-medication (SM) is an essential core element for better health outcomes. It is influenced mainly by the level and adequacy of knowledge, attitude, and appropriateness of practice (KAP) towards SM. The present study explored the level and adequacy of KAP among residents of Riyadh city, Saudi Arabia. A convenient snowball sampling method was utilized to recruit the study participants. Data were analyzed using SPSS version 27. Six hundred and eleven participants completed the questionnaire. Residents with good knowledge, positive attitude, and proper practice were 43.7%, 33.1%, and 90.0%, respectively. The level of KAP was significantly influenced by the participant’s occupation, age, gender, nationality, marital status, presence of chronic disease, and COVID-19 infection status. The mean knowledge, attitude, and practice scores observed were 5.11 (SD = 1.27), 22.28 (SD = 2.6) and 5.20 (SD = 1.29), respectively. Pearson correlation and scattered plot matrix analysis revealed a significant positive weak correlation among KAP, indicating that residents with good knowledge possess better attitude (r = 0.142, *p* < 0.001) and follow the proper practice (r = 0.256, *p* < 0.001) towards SM. Multivariate linear regression revealed a significant (*p* < 0.001) negative (β = −0.059) influence of occupation, family members working in the health sector (*p* = 0.046, β= −0.426), Body Mass Index (*p* = 0.019, β = −0.049), and physical activity (*p* = 0.018, β = −0.292) on the overall KAP score. Understanding the residents’ level of KAP towards SM would enable the health care system to identify the gap and develop a mechanism to educate the people and make them knowledgeable about SM and self-care.

## 1. Introduction

Self-Medication (SM) refers to the use of medications to treat self-diagnosed disorders without seeking medical advice [1], by using over the counter (OTC) drugs, prescription-only drugs, pharmacist-only medicines, complementary and alternative medicine [2], taking medicines on the advice of a relative and others, or consuming leftover medicines available at home [3]. SM is practiced commonly both in developing and developed countries as a part of self-care [4]. Appropriate SM has numerous advantages, including preventing, treating, and providing affordable alternatives to manage minor illnesses [5]. Contrary to this, irrational SM, which includes error in self-diagnosis, selecting incorrect drug, failing to understand drug labels and leaflets, and lack of awareness about drug interactions will lead to non-effectiveness, drug resistance, severe side effects, drug and food interactions, drug allergy, delay in actual diagnosis, overdose, and drug toxicity [6,7].

SM is a global issue that can potentially contribute to various health hazards including antibiotic resistance [8]. However, its trends differ depending on various factors, such as socioeconomic factors, medical knowledge, satisfaction, people’s perception of the disease, easy access to drugs, increased ability to manage certain illnesses through self-care, and greater availability of medicinal products [6,9]. While the objective of SM is straightforward, most people practice it without adequate knowledge, attitudes, and background, especially in countries where the public bears their health expenses on their own. Although the government and insurance companies look after health expenses in Saudi Arabia for their citizens and residents, SM practice commonly uses drugs received through family and friends, leftover medications, and OTC drugs [10,11].

SM is prevalent among populations in developing countries, and according to several reports, prescription medications can be purchased without a prescription [12,13]. Similar to most other countries, in Saudi Arabia, private sector pharmacies are the most accessible healthcare facilities. The prevalence of SM in Arab countries, including Saudi Arabia, ranges between 35% and 92% [14,15,16,17]. Various local studies have highlighted the prevalence of SM among residents of Saudi Arabia, including the use of antibiotics (34%) without a prescription, depending on advice from family and friends to use medications for self-care (68.6%) and self-use of medications to manage oral health problems (50.4%), manifesting the high prevalence and inappropriateness of SM practice [18,19,20]. Antibiotic misuse significantly threatens infectious disease control and public health in general. Lack of public awareness, knowledge, attitude, and practices about SM are potential risk factors for inappropriate drug use. Thus, researchers highlight irrational SM and related critical issues such as antibiotic resistance and potential adverse events, necessitating the imposition of healthcare initiatives to raise knowledge and awareness [21]. Most SM studies on KAP have been conducted among healthcare professionals or students, with only a few studies involving the public in Saudi Arabia [22,23,24].

The appropriateness and rationality of SM are greatly influenced by the individual’s level of knowledge, attitude, and type of practice (KAP). Studies of this kind can shed light on why patients engage in such irrational SM behavior and may aid policymakers and regulators in streamlining drug rules, updating the list of essential medicines, and addressing safety concerns with OTC medications. The proper knowledge about SM and medications used to treat self-diagnosed minor illnesses is vital in preventing health hazards, likewise, the level of attitude greatly influences SM practice and following proper practices such as storage of medications, checking expiration dates, and encouraging rational SM exercise. The Sustainable Development Goal (SGD) three of the 2030 agenda described by the United Nations (UN) recommends ensuring healthy lives and promoting well-being for all at all ages, which could be effectively achieved through public education and awareness [25]. Considering this, a study was conducted among the community of Riyadh, Saudi Arabia, to assess their KAP about SM and to investigate the association and correlation between KAP and sociodemographic variables.

## 2. Materials and Methods

### 2.1. Study Design and Population

This study was carried out from September to November 2021 among residents of Riyadh city, aged 18 years and older, to explore the knowledge, attitude, and practices towards SM. The study was approved with the registration number (02-20102021) from AlMaarefa University’s ethical board. The study location was segregated into five zones: North, South, East, West, and Central Riyadh. Those staying out of Riyadh, aged under 18 years, and residing in Riyadh on visit visas were eliminated from enrolling in this study. The purpose of the survey and the informed consent was set out at the start of an online questionnaire. Once participants read the consent form and study purpose, they could either continue to participate in the study by clicking on the “agree icon” or clicking on the “disagree icon” if they wished to not participate, thus making involvement completely voluntary. Disagreeing residents were directed to decline the participation section and exit from the survey.

### 2.2. Sample Size and Sampling Method

In 2021, the overall population of Riyadh was estimated to be about 8,175,378, according to the Saudi census department [https://portal.saudicensus.sa/portal/public/1/15/100645?type=TABLE (last accessed on 20 September 2023)]. The final minimum sample size of 385 was computed through the Raosoft sample size calculator using a formula x = Z(c/100)2r(100-r) where N is the population size, r is the fraction of responses, and Z(c/100) is the critical value for the confidence level c. We speculated a 5% level of precision, a margin of error of 5%, and a 95% confidence level to calculate the required sample size. We collected 625 responses warranting adequate representation. We mainly used a convenient sampling method to recruit the participants by visiting shopping malls, supermarkets, and other public places. The questionnaire was posted on various social media platforms (Twitter, Facebook, LinkedIn, Instagram, WhatsApp Status). Moreover, we also adopted the snowball sampling technique through personal contact to obtain more significant responses.

### 2.3. Construction, Validation and Reliability of Study Tool

An extensive review of relevant literature was carried out to construct the initial questionnaire [7,15,26,27,28,29], followed by a comprehensive revision from experts in the field of community medicine, pharmacy, and epidemiology to ensure content validity. Since the study questionnaires were adopted from previously published relevant studies, the content validity index and construct validity tests were not performed. The study tool was divided into sections as follows; the first section depicted sociodemographic details about contributors, including age, education, nationality, gender, occupation, marital status, number of children at home, family members working in the health sector (people primarily engaged in enhancing health by providing preventive, curative, promotional, or rehabilitative health care services such as a physician, nurse, pharmacist, optometrist, occupational therapist, etc.), presence of chronic disease (conditions that last one year or more and require ongoing medical attention or limit activities of daily living or both) and so on. The second section documented the participant’s knowledge about SM using seven statements. The third section was embedded with six Likert scale statements to evaluate the attitude towards SM, and the last part measured the appropriateness of SM practice using seven statements. To ensure face validity, five participants aged 20–50 years were recruited indiscriminately from each zone of Riyadh city (a total of 25 participants) to participate in the pilot study. We simplified the English terminology and decreased the number of questions as a result of the comments from the pilot respondents. Additionally, the demographic question “number of children” was modified to “a number of children staying with you” since some participants mentioned they have children but are not staying with them in Riyadh city. The pilot study responses were not included in the final data analysis. Additionally, the reliability of each questionnaire domain was calculated using the following formulae and Cronbach’s alpha factor was found to be satisfactory (knowledge; 0.75, attitude; 0.8, and practice; 0.7).

α = (K/(K − 1)) × (1 − (∑Vi)/VT) (1)

where K is the number of variables, Vi is the variance of all items, and VT is the group total variance.

### 2.4. Data Collection and Categorization of KAP Using Cut-Off Points

The final bilingual questionnaire (English and Arabic) was disseminated through social media platforms and personal contacts. Participants’ knowledge and practice regarding SM were evaluated using 7 questions on a 7-point scale. Every question was scored 1 for correct and 0 for incorrect answers. The expected maximum cumulative score was 7. The level of knowledge was categorized as good, moderate, and poor using the original Bloom’s cut-off points (Good, 80–100%; Moderate, 50–79%; and Poor, <50%) [14]. A score of 6–7 points was regarded as good, 4–5 points was regarded as moderate, and ≤3 was regarded as poor. Likewise, the participant’s practice was classified as proper (≥4 points) and improper (≤3 points). Similarly, the attitude was evaluated using six statements on a Likert scale, from strongly agree (5), agree (4), neutral (3), disagree (2), to strongly disagree (1). The minimum possible expected score was 6 and the maximum was 30. The modified Bloom’s cut-off points were used to categorize the attitude levels. A score of 24–30 (80–100%) was considered positive, 18–23 (60–79%) was neutral, and <18 (<60%) was negative.

### 2.5. Statistical Analysis

The completeness and uniformity of the collected responses were checked before subjecting them to analysis. The responses that disagreed and had incomplete demographic details were excluded from the analysis. The data were analyzed using SPSS (version 27.0). We hypothesized the discrepancy in knowledge, attitude, and practice levels among study participants. The Chi-square test was applied to determine the significance and association between dependent and independent variables; the correlation between KAP was determined using Pearson’s correlation. Multiple linear regression determined the relationship between participants’ KAP and demographic variables. A *p*-value of less than 0.05 was considered statistically significant.

## 3. Results

### 3.1. Sociodemographic Details

Altogether, 625 responses were collected, of which only 611 were included in the data analysis. We excluded the 14 responses that were incomplete and disagreed by participants. The highest number of responses were recorded from the north zone (32.7%), followed by central (18%), east (17.7%), west (16.5%), and south (15.1%) of Riyadh city. Major contribution was observed from unmarried (52.4%); females (71.4%); Saudi citizens (88.4%), and staying with family (89.7%). About 89.2% of participants had received 2 doses of COVID-19 vaccination (Table 1).

### 3.2. Resident’s Level of Knowledge, Attitude, and Practice about SM

**Figure 1:**
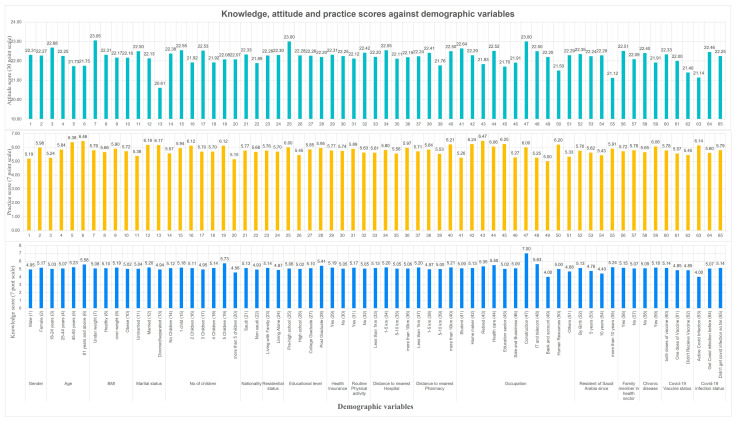
Knowledge, attitude, and practice scores against demographic variables.

The average knowledge score of residents was 5.11 (SD = 1.27) on a 7-point scale. Overall, the participants with good and moderate knowledge were almost similar, 267 (43.7%) and 277 (45.3%), respectively. About 67 (11%) displayed poor knowledge of SM (Table 2). Only the occupation significantly (*p* = 0.043) influenced the respondents’ knowledge level. Residents who are working in construction (7 points), IT and telecommunication (5.63 points), and the health sector (5.5 points) significantly displayed a good level of knowledge about SM compared to the residents who work in banks and accounts (4 points) and human resources (5 points) (Figure 1). Similarly, the mean attitude score was 22.28 (SD = 2.6) on a 30-point scale. More than half of the respondents, 391 (64%) and 18 (3%), have displayed a neutral and negative attitude towards SM, respectively. Nevertheless, 202 (33.1%) of the participants have shown a positive attitude toward SM Table 2. Association between demographic variables and adequacy of knowledge, attitude, and practice towards self-medication.

Age, marital status, nationality, and COVID-19 infection status significantly influenced the participants’ attitudes (Table 2). Residents aged 18–24 years showed a significantly (*p* = 0.001) better attitude score (22.7 points) compared (21.7 points) to residents who were 61 years old and above. Likewise, unmarried respondents had a significantly (*p* < 0.001) better attitude score (22.5 points) compared to (20.6) divorced residents. Saudi nationals exhibited a significantly (*p* = 0.041) more excellent attitude score (22.3 points) compared to (21.9 points) non-Saudi residents. Comparably, respondents who recovered from COVID-19 infection displayed a significantly (*p* = 0.001) superior attitude score (22.5 points) compared to respondents (21.1 points) who had an active COVID-19 infection (Figure 1). Most respondents (*n* = 550,90%) exhibited proper practice toward SM. Gender, age, marital status, occupation, and presence of chronic disease significantly impacted the magnitude of practice toward SM (Table 2). The female participants showed significantly (*p* < 0.001) better practice scores (6 points) compared to their male counterparts (5.2 points). Residents above 61 years old displayed significantly (*p* < 0.001) superior practice scores (6.5 points) compared to younger adults (5.2 points). Married and divorced residents showed significantly (*p* < 0.001) more outstanding practice scores (6.2 points) compared to unmarried (5.4 points). Residents who were suffering from any long-term or chronic disease exhibited significantly (*p* = 0.015) better practice scores (6.1 points) compared to residents (5.7 points) who were healthy (Figure 1).

### 3.3. Basic Information about SM Knowledge, Attitudes, and Practice

**Figure 2:**
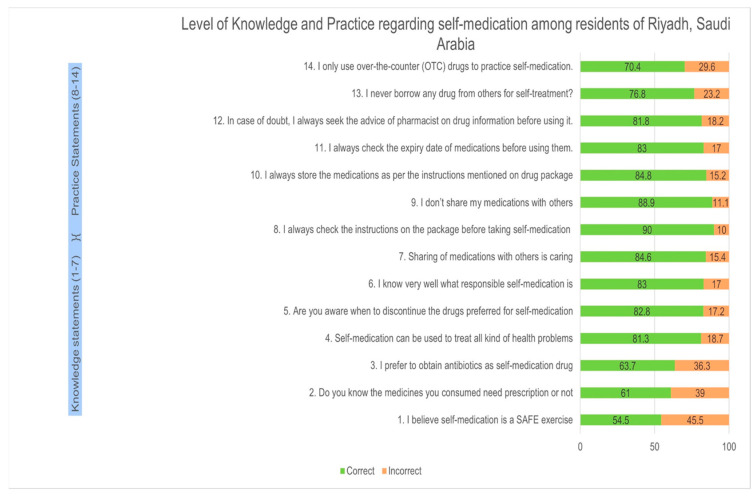
Pattern of correct and incorrect answers about the knowledge and practice of SM.

**Figure 3:**
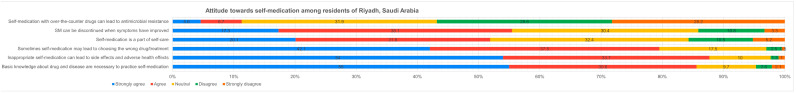
Attitude towards self-medication among residents of Riyadh, Saudi Arabia.

This study noted that more than 50% of respondents correctly identified the basic knowledge and practices of SM. However, it was found that less than 80% of respondents correctly answered the question on obtaining antibiotics for SM, information about OTC and prescription medications and characterizing SM as a safe exercise. Similarly, about 30% of participants still follow the improper way of SM practice, such as borrowing drugs and using prescription drugs for SM (Figure 2). In terms of attitude, more than 50% of respondents agree with statements such as “Basic knowledge about drugs and diseases are necessary to practice SM”, “inappropriate SM can lead to adverse health effects”, and “sometimes SM can lead to the selection of the wrong drug”, “SM is part of self-care”, and “discontinuing SM once symptoms have improved”. More than 50% of participants disagree with the statement that “SM with OTC drugs can lead to antimicrobial resistance” (Figure 3).

### 3.4. Correlation between Knowledge, Attitude, and Practice towards SM

**Figure 4:**
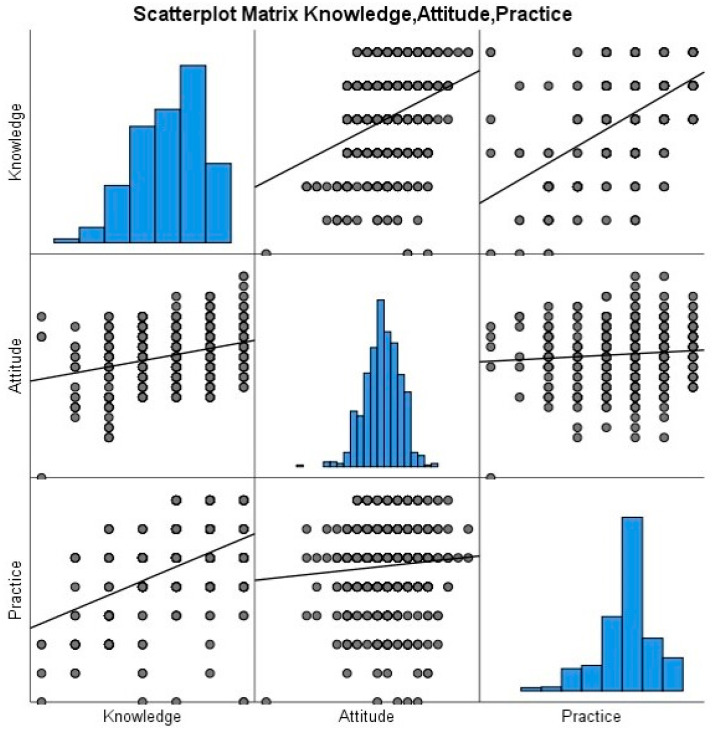
Scattered plot matrix depicted the nature of correlation among knowledge, attitude, and practice about SM.

The diagonal histograms in the scattered plot matrix show the normal data distribution in knowledge, attitude, and practice domains. The fit line in the scattered plot indicates a positive correlation between knowledge with attitude and practice, indicating that as knowledge increases the level of attitude and appropriateness of practice increase, and vice versa. However, the fit line of scattered plot in attitude and practice is not steep, indicating low positive correlation among them (Figure 4). Further significance of these correlations is confirmed by applying the Pearson correlation analysis. A correlation coefficient (r) value of less than 0.39 is considered a weak correlation. A significant positive weak (r = 0.142) correlation was observed between knowledge with attitude (*p* < 0.001) and knowledge with practice (r = 0.256, *p* < 0.001) about SM. This shows that the participants with a good level of knowledge about SM will have a positive attitude and follow proper practices and vice versa. However, no correlation was observed between attitude and practice as an r value less than 0.1 indicates no correlation among the variables (Table 3).

### 3.5. Factors Affecting KAP Score-Multiple Linear Regression Analysis

The scores of knowledge, attitude, and practice are dependent variables. The multiple linear regression was performed on the factors influencing the total KAP score, such as gender, age, education, occupation, BMI, physical activity, and family members in the health sector. Table 4 depicts the results of the independent variables that significantly (*p* < 0.05) impacted the overall score of knowledge, attitude, and practice about SM. The resident’s occupation showed a significant (*p* < 0.001) negative impact (β= −0.059) on the knowledge score. Similarly, BMI (β= −0.049) and physical activity (β= −0.292) significantly negatively impacted the practice scores. Surprisingly, respondents with family members working in the health sector significantly negatively impacted their attitude. Conversely, participants’ age, gender, and education positively impacted knowledge and practice scores. The other independent variables such as marital status, number of children, nationality, and distance to nearest healthcare facility did not show any significant impact on the scores of knowledge, attitude, and practice (Table 4).

## 4. Discussion

Although SM is a part of self-care and recommended by the World Health Organization (WHO), its effectiveness depends on appropriateness and sensible application. Moreover, the appropriateness and rationality of SM practice are influenced mainly by the participant’s level of knowledge, attitude, and type of practice regarding SM. There are numerous health benefits of SM, but it depends on how it is practiced and who practices it.

In this study, participants have displayed sufficient overall knowledge, good attitude, and proper practices towards SM. The previous studies carried out in Riyadh city to explore the pattern, prevalence, and predictors of SM were “Self-medication in central Saudi Arabia: community pharmacy consumers perspective” in 2011 [12] and “Self-medication practice among patients in a public health care system” in 2009 [14]. Residents visiting community pharmacies were selected in the 2011 study, whereas people visiting primary healthcare centers were targeted in the 2009 study. In contrast, this study included residents from all over Riyadh city. The study conducted in Riyadh in 2009 included 500 adult patients attending primary health care, in which 55.8% were male, whereas the study conducted in 2011 collected data from 538 participants, in which 73% were male. The present study collected data from 611 participants, higher than those earlier studies, in which 71.4% were females.

The findings show that 43.7% of respondents had good knowledge regarding SM, which is higher than the earlier study conducted in Riyadh on community pharmacy consumers (30.3%) [12]. In contrast, studies conducted in the Ethiopian community [24] (83.4%) and Haikou, China [7] (63.48%), observed a higher number of participants with a good level of knowledge. Similarly, the present study noticed a higher number (33.1%) of respondents with positive attitudes compared to the residents of Haikou (16.6%), China. [7] However, an earlier study conducted in Riyadh (62.6%) among community pharmacy consumers [12] showed a high number of respondents with a positive attitude. This study participants with a neutral attitude were 64%, comparable to Haikou (60.7%), China, findings [7]. In contrast, present participants with proper practice towards SM were 90%, unlike the observations of Haikou (95%), China [7]. Present study demonstrated a significant (*p* = 0.043) impact of occupation on the level of knowledge regarding SM. A similar observation was noted in an earlier study conducted in Riyadh in 2011 [12] (*p* = 0.044). In the present study, we noticed the significant impact of nationality, marital status, age, and COVID-19 infection status on the level of attitude, unlike earlier findings in Riyadh among community pharmacy consumers [12], where no significant impact of any variable was noted.

Based on the univariant analysis, present findings indicated the significant impact of occupation on the adequacy of knowledge, unlike a study conducted in Haikou, China, which observed a significant impact of age, medical insurance, and education level. Additionally, participants’ nationality, marital status, and COVID-19 infection status significantly impacted the attitude towards SM, unlike findings from Haikou, China, which noted the impact of education, and occupation on the appropriateness of attitude [7]. The correlation between knowledge and attitude was weak, but it was significantly positive, indicating that as the knowledge about SM increases, the level of attitude also increases. Similarly, a weak positive significant correlation was observed between knowledge and practice about SM, which means as the knowledge about SM increases, the appropriateness about SM practice increases.

Additionally, we observed a significant impact of gender, age, marital status, occupation, and the presence of chronic disease on the appropriateness of practice, which are in line with the findings of China [7], which noted the impact of age, medical insurance, education, employment and occupation, etc. Moreover, multivariant linear regression analysis was performed to predict the confounding factors affecting KAP towards SM. This study noted a significant positive influence of age and education on knowledge score, which is in line with the findings of Haikou, China, where the level of education was positively correlated with knowledge. In contrast, age was negatively associated with knowledge scores in the same study. Similarly, we observed the significant positive impact of education on practice scores; similar findings were noted in a study in Haikou, China [7]. Overall, COVID-19 infection/recovery and being Saudi national showed to have a positive impact on the level of attitude towards SM. Similarly, age and education showed significantly positive impact on knowledge and practice towards SM. Hence, we assume, that being young, educated, Saudi national, and a previous COVID-19 infection status has a positive impact on KAP towards SM.

More than half of the participants (55.5%) considered SM as a safe practice, which is comparatively higher (33.5%) than findings from residents of Hail [26], Saudi Arabia, but lower compared to the findings of urban Pondicherry (66.6%), India [27]. The findings of this study revealed that more than 80% of residents check the expiry date of medication before using them, similar to the findings of Hail residents [26], Saudi Arabia (85.23%), and higher than the findings of the Ethiopian [24] community (63.7%). About 80% of respondents agree that SM may lead to an incorrect choice of therapy or drug. A similar outcome was mentioned in a study conducted in Hail [26] (83.4%), Saudi Arabia. More than 60% of participants knew that the medicine they used was prescription or OTC, similar to the findings of an earlier study conducted in Riyadh [12], community pharmacy consumers (68.2%), and the adult population of the eastern province (68.6%), Saudi Arabia [28]. The reason for better awareness regarding this statement could be the participant’s study location (pharmacy setting), making them more responsible while responding. Consulting pharmacists for more information regarding drugs used for self-care was stated by about 80% of participants, which is higher compared to the findings from earlier studies conducted in Riyadh, 2011 [12] (37.3%), the adult population of the eastern province, Saudi Arabia [28] (42.29%) and Hail [26], Saudi Arabia (70%). About 90% of participants mentioned that they do not share their medications with others, indicating their responsible behavior and best practice, which is higher than the Ethiopian [24] community (74.7%). Similarly, 90% of respondents stated reading drug instructions on drug packages before using them for self-care, which is a rational practice. Findings from other studies show the low practice of reading drug pamphlets and instructions such as Hail [26], Saudi Arabia (30%), the adult population of the eastern province [28], Saudi Arabia (23.49%), and Riyadh community pharmacy [12] consumers (60%) during 2011.

Irrational SM could lead to adverse health consequences, incorrect diagnosis, increasing the risk of disease exacerbation and increase the burden on the healthcare system. The knowledge, attitude, and practice regarding SM significantly influence the rationality and appropriateness of SM practice. Overall, residents of Riyadh city, Saudi Arabia, have displayed a desirable and satisfactory level of knowledge, attitudes, and practice towards SM. The strategies could be developed to improve the public knowledge and awareness about SM, which will have a direct impact on their health outcome. This includes but is not limited to awareness programs at public places, counselling by community pharmacists, advertisements, and pamphlets.

## 5. Conclusions

Overall, the residents of Riyadh city have demonstrated an adequate level of knowledge, attitude, and practice scores regarding SM, which would directly impact the rationality and responsibility towards SM practice. Age, education, gender, BMI, occupation, physical activity, and family members working in the healthcare sector were found to have a significant impact on the KAP of residents towards SM. One of the key elements to promote well-being to all ages is through education and awareness. The findings would serve to highlight the gaps in KAP pertaining to self-medication among the population. Consequently, it would be possible to formulate strategies aimed at enhancing the general population’s awareness and knowledge regarding self-medication, thereby directly influencing their health outcomes.

## 6. Strengths and Limitations

The present study has several strengths that are worth mentioning. We collected 611 complete responses, although a minimum sample of 385 was adequate. Participants were recruited from all the areas of Riyadh city, although the majority were from the north of Riyadh. We excluded the participation of AlMaarefa university students because they have recently participated in a similar study. Additionally, residents residing on visit visas were excluded from the participation due to their limited stay in the country and negligible impact on the healthcare system of the country.

One of the limitations was approaching the participants through a convenient snowball sampling technique. An online survey was distributed through personal contacts, social media platforms (Twitter, Facebook, LinkedIn, Instagram, WhatsApp), and visiting selected public places (shopping malls, supermarkets, public parks). This study was conducted in Riyadh city; hence, the study observations cannot be generalized to the entire Saudi population. The study duration was limited to three months duration. A larger sample study with a longer duration is needed to further validate these findings.

## Figures and Tables

**Table 1:** Details about demographic characteristics of participants.

Demographic Variables		Demographic Characteristics (N)	Percentage
Gender	Male	175	28.6
	Female	436	71.4
Age	18–24 years	231	37.8
	25–44 years	221	36.2
	45–60 years	135	22.1
	61 years and above	24	3.9
BMI	Underweight	38	6.2
	Healthy	241	39.4
	Overweight	191	31.3
	Obese	141	23.1
Marital status	Unmarried	320	52.4
	Married	273	44.7
	Divorced/separated	18	2.9
Number of children at home	No Children	217	35.5
	1 child	104	17
	2 Children	98	16
	3 Children	76	12.4
	4 Children	63	10.3
	5 Children	26	4.3
	>5 children	27	4.4
Nationality	Saudi	540	88.4
	Non-Saudi	71	11.6
Residential status	Living with Family	548	89.7
	Living Alone	63	10.3
Education level	Pre-high school	16	2.6
	High school	169	27.7
	College Graduate	360	58.9
	Postgraduate	66	10.8
Health insurance	Yes	281	46
	No	330	54
Physical activity	Yes	290	47.5
	No	321	52.5
Distance to the nearest hospital	Less than 1 km	93	15.2
	1–5 km	182	29.8
	5–10 km	160	26.2
	More than 10 km	176	28.8
Distance to nearest pharmacy	Less than 1 km	350	57.3
	1–5 km	213	34.9
	5–10 km	34	5.6
	More than 10 km	14	2.3
Occupation	Student	200	32.7
	Homemaker	79	12.9
	Retired	66	10.8
	Health care	66	10.8
	Education sector	81	13.2
	Sale and Business	22	3.6
	Construction	1	0.2
	IT and telecom	8	1.3
	Bank and account	5	0.8
	Human Resources	10	1.6
	Others	73	11.9
Residing in Saudi Arabia since	By birth	542	88.7
	5 years	29	4.7
	10 years	7	1.1
	More than 10 years	33	5.4
Family member in the health sector	Yes	272	44.5
	No	339	55.4
Chronic disease	No	449	73.5
	Yes	159	26
COVID-19 vaccine status	Two doses of vaccine	545	89.2
	One dose of vaccine	46	7.5
	Have not received vaccine	20	3.3
COVID-19 infection status	Active	7	1.1
	Recovered	131	21.4
	Have not infected by COVID-19 so far	473	77.4

**Table 2:** Association between demographic variables and adequacy of knowledge, attitude and practice towards self-medication.

Demographic Variables		Adequacy of Knowledge (N = 611)				Level of Attitude (N = 611)				Type of Practice (N = 611)		
		Poor (%)	Moderate (%)	Good (%)	*p* Value	Negative (%)	Neutral (%)	Positive (%)	*p* Value	Improper (%)	Proper (%)	*p* Value
Gender	Male	25 (4.1)	80 (13.1)	70 (11.5)	0.198	7 (1.1)	110 (18)	58 (9.5)	0.614	31 (5.1)	144 (23.6)	<0.001 *
	Female	42 (6.9)	197 (32.2)	197 (32.2)		11 (1.8)	281 (46)	144 (23.6)		30 (4.9)	406 (66.4)	
Age	18–24 years	31 (5.1)	99 (16.2)	101 (16.5)	0.264	3 (0.5)	132 (21.6)	96 (15.7)	0.001 *	35 (5.7)	196 (32.1)	<0.001 *
	25–44 years	24 (3.9)	109 (17.8)	88 (14.4)		5 (0.8)	149 (24.4)	67 (11)		23 (3.8)	198 (32.4)	
	45–60 years	12 (2)	58 (9.5)	65 (10.6)		9 (1.5)	96 (15.7)	30 (4.9)		2 (0.3)	133 (21.8)	
	61 years and above	zero	11 (1.8)	13 (2.1)		1 (0.2)	14 (2.3)	9 (1.5)		1 (0.2)	23 (3.8)	
BMI	Underweight	4 (0.7)	18 (2.9)	16 (2.6)	0.565	zero	20 (3.3)	18 (2.9)	0.322	3 (0.5)	35 (5.7)	0.346
	Healthy	29 (4.7)	103 (16.8)	109 (17.8)		7 (1.2)	148 (24.2)	86 (14.1)		30 (4.9)	211 (34.5)	
	Overweight	19 (3.1)	82 (13.4)	90 (17.7)		6 (1)	128 (20.9)	57 (9.3)		14 (2.3)	177 (29)	
	Obese	15 (2.5)	74 (12.1)	52 (8.5)		5 (0.8)	95 (15.5)	41 (6.7)		14 (2.3)	127 (20.8)	
Marital Status	Unmarried	43 (7)	137 (22.4)	140 (22.9)	0.293	5 (0.8)	194 (31.8)	121 (19.8)	<0.001 *	47 (7.7)	273 (44.7)	<0.001 *
	Married	22 (3.6)	131 (21.4)	120 (19.6)		10 (1.6)	186 (30.4)	77 (12.6)		13 (2.1)	260 (42.6)	
	Divorced/separated	2 (0.3)	9 (1.5)	7 (1.1)		3 (0.5)	11 (1.8)	4 (0.7)		1 (0.2)	17 (2.8)	
Number of children at home	No Children	27 (4.4)	94 (15.4)	96 (15.7)	0.532	7 (1.2)	132 (21.6)	78 (12.8)	0.928	29 (4.7)	188 (30.8)	0.095
	1 child	8 (1.3)	51 (8.3)	45 (7.4)		4 (0.7)	63 (10.3)	37 (6.1)		6 (1)	98 (16)	
	2 Children	12 (2)	43 (7)	43 (7)		3 (0.5)	67 (11)	28 (4.6)		7 (1.1)	91 (14.9)	
	3 Children	11 (1.8)	33 (5.4)	32 (5.2)		1 (0.2)	50 (8.2)	25 (4.1)		8 (1.3)	68 (11.1)	
	4 Children	4 (0.7)	32 (5.2)	27 (4.4)		2 (0.3)	45 (7.4)	16 (2.6)		6 (1)	57 (9.3)	
	5 Children	zero	11 (1.8)	15 (2.5)		zero	17 (2.8)	9 (1.5)		zero	26 (4.3)	
	>5 children	5 (0.8)	13 (2.1)	9 (1.5)		1 (0.2)	17 (2.8)	9 (1.5)		5 (0.8)	22 (3.6)	
Nationality	Saudi	58 (9.5)	243 (39.8)	239 (39.1)	0.718	17 (2.8)	336 (55)	187 (30.6)	0.041 *	57 (9.3)	483 (79.1)	0.193
	Non-Saudi	9 (1.5)	34 (5.6)	28 (4.6)		1 (0.2)	55 (9)	15 (2.5)		4 (0.7)	67 (11)	
Residential status	Living with Family	58 (9.5)	246 (40.2)	244 (39.9)	0.413	15 (2.5)	350 (57.3)	183 (30)	0.613	55 (9)	493 (80.7)	0.898
	Living Alone	9 (1.5)	31 (5.1)	23 (3.8)		3 (0.5)	41 (6.7)	19 (3.1)		6 (1.0)	57 (9.3)	
Educational qualification	Primary school	2 (0.3)	7 (1.2)	7 (1.2)	0.809	zero	10 (1.6)	6 (1.0)	0.626	2 (0.3)	14 (2.3)	0.292
	High school	20 (3.3)	80 (13.1)	69 (11.3)		6 (1.0)	104 (17)	59 (9.7)		23 (3.8)	146 (23.9)	
	College Graduate	39 (6.4)	165 (27)	156 (25.5)		8 (1.3)	237 (38.8)	115 (18.8)		30 (4.9)	330 (54)	
	Postgraduate	6 (1.0)	25 (4.1)	35 (5.7)		4 (0.7)	40 (6.5)	22 (3.6)		6 (1.0)	60 (9.8)	
Health insurance	Yes	28 (4.6)	123 (21.3)	130 (21.3)	0.463	8 (1.3)	179 (29.3)	94 (15.4)	0.976	24 (3.9)	257 (42)	0.272
	No	39 (6.4)	154 (25.2)	137 (22.4)		10 (1.6)	212 (34.7)	108 (17.7)		37 (6.1)	293 (48)	
Physical activity	Yes	32 (5.2)	122 (20)	136 (22.3)	0.273	9 (1.5)	192 (31.4)	89 (14.6)	0.495	23 (3.8)	267 (43.7)	0.108
	No	35 (5.7)	155 (25.4)	131 (21.4)		9 (1.5)	199 (32.6)	113 (18.5)		38 (6.2)	283 (46.3)	
Distance to nearest hospital	Less than 1 km	9 (1.5)	45 (7.4)	39 (6.4)	0.231	3 (0.5)	57 (9.3)	33 (5.4)	0.606	11 (1.8)	82 (13.4)	0.410
	1–5 km	12 (2)	91 (14.9)	79 (12.9)		4 (0.7)	115 (18.8)	63 (10.3)		17 (2.8)	165 (27)	
	5–10 km	23 (3.8)	64 (10.5)	73 (11.9)		8 (1.3)	101 (16.5)	51 (8.3)		20 (2.3)	140 (22.9)	
	More than 10 km	23 (3.8)	77 (12.6)	76 (12.4)		3 (0.5)	118 (19.3)	55 (9)		13 (2.1)	163 (26.7)	
Distance to nearest pharmacy	Less than 1 km	33 (5.4)	157 (25.7)	160 (26.1)	0.587	10 (1.6)	227 (37.2)	113 (18.5)	0.520	38 (6.2)	312 (51.1)	0.777
	1–5 km	27 (4.4)	101 (16.5)	85 (13.9)		5 (0.8)	134 (21.9)	74 (12.1)		18 (2.9)	195 (31.9)	
	5–10 km	6 (1)	13 (2.1)	15 (2.5)		3 (0.5)	21 (3.4)	10 (1.6)		4 (0.7)	30 (4.9)	
	More than 10 km	1 (0.2)	6 (1.0)	7 (1.1)		zero	9 (1.5)	5 (0.81)		1 (0.2)	13 (2.1)	
Occupation	Student	25 (4.1)	84 (13.7)	91 (14.9)	0.043 *	5 (0.8)	112 (18.3)	83 (13.6)	0.214	31 (5.1)	169 (27.7)	0.003 *
	Home maker	8 (1.3)	34 (5.6)	37 (6.1)		1 (0.2)	54 (8.8)	24 (3.9)		3 (0.5)	76 (12.4)	
	Retired	3 (0.5)	31 (5.1)	32 (5.2)		2 (0.3)	48 (7.9)	16 (2.6)		1 (0.2)	65 (10.6)	
	Health care	3 (0.5)	27 (4.4)	36 (5.9)		3 (0.5)	38 (6.2)	25 (4.1)		4 (0.7)	62 (10.1)	
	Education sector	9 (1.5)	43 (7)	29 (4.7)		6 (1)	54 (8.8)	21 (3.4)		3 (0.5)	78 (12.8)	
	Sale and Business	2 (0.3)	13 (2.1)	7 (1.1)		1 (0.2)	15 (2.5)	6 (1)		5 (0.8)	17 (2.8)	
	Construction	zero	zero	1 (0.2)		zero	1 (0.2)	zero		zero	1 (0.2)	
	IT and telecom	zero	3 (0.5)	5 (0.8)		zero	5 (0.8)	3 (0.5)		1 (0.2)	7 (1.1)	
	Bank and account	2 (0.3)	3 (0.5)	zero		zero	3 (0.5)	2 (0.3)		1 (0.2)	4 (0.7)	
	Human Resources	zero	7 (1.1)	3 (0.5)		zero	8 (1.3)	2 (0.3)		1 (0.2)	9 (1.5)	
	Others	15 (2.5)	32 (5.2)	26 (4.3)		zero	53 (8.7)	20 (3.3)		11 (1.8)	62 (10.2)	
Residing in Saudi Arabia since	By Birth	57 (9.3)	245 (40.1)	240 (39.3	0.518	16 (2.6)	339 (55.5)	187 (30.6)	0.330	54 (8.8)	488 (79.9)	0.735
	5 years	5 (0.8)	15 (2.5)	9 (1.5)		1 (0.2)	19 (3.1)	9 (1.5)		4 (0.7)	25 (4)	
	10 years	2 (0.3)	3 (0.5)	2 (0.3)		zero	6 (1.0)	1 (0.2)		zero	7 (1.1)	
	More than 10 years	3 (0.5)	14 (2.3)	16 (2.6)		1 (0.2)	27 (4.4)	5 (0.8)		3 (0.5)	30 (4.9)	
Family member in health sector	Yes	24 (3.9)	124 (20.3)	124 (20.3)	0.292	6 (1.0)	170 (27.8)	96 (15.7)	0.402	28 (4.6)	244 (39.9)	0.819
	No	43 (7.0)	153 (25)	143 (23.4)		12 (2.0)	221 (36.1)	106 (17.3)		33 (5.4)	306 (50)	
Chronic disease	No	54 (8.8)	198 (32.4)	197 (32.2)	0.245	10 (1.6)	282 (46.1)	157 (25.7)	0.054	53 (8.7)	396 (64.8)	0.015 *
	Yes	12 (2.0)	78 (12.8)	69 (11.2)		8 (1.3)	108 (17.7)	43 (7.0)		8 (1.3)	151 (24.7)	
COVID-19 vaccine status	Got two doses	59 (9.7)	243 (39.8)	243 (39.8)	0.767	16 (2.6)	344 (56.3)	185 (30.3)	0.152	52 (8.5)	493 (80.7)	0.302
	Got first dose	6 (1.0)	23 (3.8)	17 (2.8)		zero	34 (5.6)	12 (2.0)		5 (0.8)	41 (6.7)	
	Didn’t receive	2 (0.3)	11 (1.8)	7 (1.1)		2 (0.3)	13 (2.1)	5 (0.8)		4 (0.7)	16 (2.6)	
COVID-19 infection status	Active	2 (0.3)	5 (0.8)	zero	0.144	2 (0.3)	2 (0.3)	3 (0.5)	0.001 *	zero	7 (1.1)	0.654
	Recovered	14 (2.3)	63 (10.3)	54 (8.8)		3 (0.5)	78 (12.8)	50 (8.2)		14 (2.3)	117 (19.1)	
	No COVID-19 infection so far	51 (8.3)	209 (34.2)	213 (34.9)		13 (2.1)	311 (50.9)	149 (24.4)		47 (7.7)	426 (69.7)	

* Indicates significance with *p* value < 0.05.

**Table 3:** Pearson correlation between knowledge, attitude, and practices towards SM.

Variable	Knowledge		Attitude		Practice	
	Correlation Coefficient (r)	*p* Value	Correlation Coefficient (r)	*p* Value	Correlation Coefficient (r)	*p* Value
Knowledge	----	----	0.142 **	<0.001	0.256 **	<0.001
Attitude			----	----	0.001	0.974
Practice					----	----

** Significant correlation at the 0.01 level (2-tailed).

**Table 4:** Multiple linear regression analysis of factors affecting participant’s knowledge, attitude, and practice towards SM.

Independent Variable	Regression Coefficient β	Standard Error	Standardized Regression Coefficient β	t	*p*-Value
Knowledge *					
Constant term	4.274	0.309	---	13.823	0.000
Age **	0.166	0.061	0.112	2.725	0.007
Education **	0.198	0.080	0.103	2.467	0.014
Occupation **	−0.059	0.017	−0.152	−3.561	<0.001
Practice *					
Constant term	4.092	0.700	---	5.845	0.000
Age **	0.552	0.079	0.293	7.007	<0.001
Gender **	0.816	0.139	0.226	5.863	<0.001
Education **	0.252	0.095	0.102	2.640	0.009
BMI **	−0.049	0.021	−0.098	−2.351	0.019
Physical activity **	−0.292	0.124	−0.089	−2.366	0.018
Attitude *					
Constant term	22.941	0.349		65.827	0.000
Member in Health sector **	−0.426	0.213	−0.081	−1.996	0.046

*—Continuous Variable; **—Categorical Variable.

## Data Availability

All relevant data has been included in the manuscript.

## Abbreviations

SM	Self-medication
OTC	Over-the-counter drugs
WHO	World health organization
KAP	Knowledge, attitude, and practice
